# Durability and Microstructural Evaluation of Geopolymer Mortars Exposed to Sulphuric Acid Using Industrial By-Product Fillers

**DOI:** 10.3390/polym17172310

**Published:** 2025-08-26

**Authors:** Ouiame Chakkor

**Affiliations:** Civil Engineering Department, Faculty of Engineering, İstanbul Aydın University, İstanbul 34295, Turkey; ouiamechakkor@aydin.edu.tr

**Keywords:** geopolymer, metakaolin, red mud, industrial by-products, sulphuric acid resistance, microstructure, durability, mechanical properties

## Abstract

Rapid urbanization and industrialization have increased atmospheric pollution, particularly via sulfur oxides (SO_x_) that form sulfuric acid and accelerate the degradation of cementitious materials. While Portland-cement systems have been widely studied, less is known about the acid resistance of geopolymer mortars. This study investigates the durability and microstructural evolution of metakaolin–red mud geopolymer mortars incorporating limestone, marble, and basalt powders as partial sand replacements (5, 10, and 15 wt %). Specimens were immersed in 3% H_2_SO_4_ for 30, 60, and 90 days, with performance evaluated via compressive and flexural strength, weight loss, and ultrasonic pulse velocity (UPV), alongside scanning electron microscopy (SEM), X-ray diffraction (XRD), and Fourier transform infrared spectroscopy (FTIR). After 90 days, the optimal basalt-filled mix (15 wt %) retained 84% of its initial compressive strength (46.8 MPa), compared with 61% for the control; mass loss decreased from 6.4% (control) to 3.2%, and UPV degradation was reduced by 35%. Microstructural analyses indicate denser gel phases and reduced microcracking in basalt- and marble-filled mixes. These results demonstrate that industrial by-product fillers can significantly improve sulfuric-acid resistance while supporting more sustainable binder technology.

## 1. Introduction

Soil pollution arising from industrial waste, sewage discharges, fuel combustion, and agricultural inputs (fertilizers/pesticides) has become a significant research focus. From a civil engineering standpoint, microbially polluted soils are hazardous to nearby structures because they accelerate concrete corrosion, material degradation, and shorten service life [1,2,3,4,5]. To mitigate these risks and reduce environmental burdens, aluminosilicate precursors—such as fly ash, red mud, metakaolin, and ground-granulated blast-furnace slag—are activated with alkaline solutions to form inorganic binders (geopolymer mortars). Given the chemical aggressivity of many service environments, the mechanical and durability performance of the resulting structures is critical to long-term reliability [6,7].

While the mechanical strength of geopolymer mortars can be broadly comparable to that of ordinary Portland cement (OPC), their manufacturing-related CO_2_ emissions are substantially lower than those associated with OPC production [8]. Consequently, metakaolin-, fly-ash-, red-mud-, and slag-based geopolymers are increasingly explored as partial or full replacements for Portland cement to lessen environmental impact [9]. Many studies report that cement manufacturing accounts for ~9% of global CO_2_ emissions, underscoring the need for lower-carbon binders.

Acidic effluents are common in industrial and wastewater contexts; phosphogypsum sites, solid-waste facilities, and electroplating storage areas often produce highly acidic soils (pH < 3) with elevated heavy-metal contents, commonly including Pb^2+^ [10,11,12,13]. Wastewater-borne bacteria generate hydrogen sulfide (H_2_S), which is biologically oxidized to sulfuric acid (H_2_SO_4_), a key driver of biogenic concrete corrosion. In sewage systems, sulfur-oxidizing bacteria—particularly Thiobacillus thiooxidans—oxidize H_2_S to H_2_SO_4_ under aerobic conditions, lowering concrete surface pH and inducing formation of expansive gypsum (CaSO_4_·2H_2_O) and ettringite (Ca_6_Al_2_(SO_4_)_3_(OH)_12_·26H_2_O) [10,11,12,13,14,15]. The acid attacks calcium hydroxide and calcium–silicate–hydrate (C–S–H) in OPC, causing cracking, spalling, and loss of integrity [13,14,16,17,18,19]. In wastewater infrastructure, this biogenic corrosion accelerates the deterioration of conventional concretes; geopolymer alternatives with improved acid resistance could meaningfully extend the service life of maintenance holes, sewer pipelines, and industrial drains.

The term “geopolymer,” introduced by Davidovits in 1978, refers to binders comprising Si–O–Al frameworks produced by reacting alkaline activators with aluminosilicate sources [20]. Geopolymers are noted for high acid and thermal resistance [20,21], lower energy demand and cost, reduced shrinkage and creep, enhanced durability [22,23], and rapid strength development [24]. Nonetheless, alkali-activated systems can exhibit higher autogenous/drying shrinkage than OPC, and highly alkaline, Ca-rich formulations may be susceptible to alkali–silica reaction (ASR). These risks can be mitigated through activator optimization (silicate modulus/alkali dosage), limiting Ca-rich precursors, selecting low-reactivity aggregates, and employing shrinkage-control measures; recent studies—including high-volume waste-glass geopolymers—document these behaviors and mitigation strategies [25,26].

Geopolymer mortars are commonly prepared with metakaolin (MK)—obtained by calcining kaolin at ~600–900 °C—as a primary aluminosilicate source, often combined with other industrial by-products such as red mud and slag [27,28,29,30]. MK–red-mud (MK–RM) binders have been investigated for acid-resistant mortars, with performance governed by precursor chemistry and activator regime. Prior work suggests that CaCO_3_-bearing fillers (limestone/marble) can densify the matrix yet may promote gypsum formation under H_2_SO_4_ exposure. In contrast, basalt powder acts mainly as a siliceous filler whose influence depends on dosage and fineness [31,32]. However, most studies vary the base binder (e.g., FA/GGBS instead of MK–RM) or examine a single filler in isolation without a unified acid-exposure protocol. As a result, direct, side-by-side comparisons within the same MK–RM binder—replacing river sand with limestone, marble, and basalt under identical mix design, curing, and sulfuric-acid exposure—and evaluated with consistent metrics (strength retention at 30/60/90 days, mass loss/UPV, and correlated SEM/XRD/FTIR) remain scarce [33,34].

Red mud itself is a multiphase residue rich in crystalline phases such as boehmite (γ-AlOOH), corundum (α-Al_2_O_3_), hematite (Fe_2_O_3_), and perovskite (CaTiO_3_), along with quartz/goethite and minor titanates. Many of these phases exhibit limited reactivity during alkali activation and, therefore, function as internal fillers that influence packing and pore structure—distinct from externally added fillers [35,36]. In parallel, numerous studies have used limestone, marble, and basalt powders to partially or fully replace sands due to their high density and their ability to fill micro- and meso-voids, thereby refining pore structure [37,38]. The present work selects these powders because they are widely available by-products with suitable fineness and because their chemistry and particle characteristics can meaningfully affect geopolymer microstructure and acid resistance [39,40].

In this study, metakaolin and red mud were used as aluminosilicate precursors to produce geopolymer mortars activated with a sodium silicate (SS)–sodium hydroxide (SH) solution (Na_2_SiO_3_/NaOH = 2:1, 12 M NaOH). To improve acid resistance and promote sustainable material reuse, industrial by-product powders—limestone, marble, and basalt—were incorporated as partial replacements for natural river sand at 5, 10, and 15 wt.%. The experimental program involved preparing and curing all mixes under identical conditions, followed by immersion in 3% sulfuric acid for 30, 60, and 90 days at ambient temperature. Durability was evaluated through compressive and flexural strength retention, mass loss, and ultrasonic pulse velocity measurements, while microstructural changes were analyzed using scanning electron microscopy (SEM), X-ray diffraction (XRD), and Fourier transform infrared spectroscopy (FTIR).

## 2. Materials and Methods

### 2.1. Raw Materials

Metakaolin (MK) was obtained from Astim Mining Company (Izmir, Türkiye) with a median particle size (d_50_) of 5.4 µm and a Blaine specific surface area of 14,200 cm^2^/g. Red mud (RM) was sourced from the Seydişehir Aluminum Plant (Konya, Türkiye), a Bayer process by-product, oven-dried at 105 °C for 24 h, and sieved to pass 75 µm; it had a median particle size of 12.6 µm. Limestone powder (LP) and marble powder (MP) were supplied by local quarries in Afyon, Türkiye, and basalt powder (BP) was obtained from a quarry in Kayseri, Türkiye. Their particle size distributions were measured via laser diffraction (Mastersizer 3000, Malvern Instruments) Mastersizer 3000 (Malvern Instruments Ltd., Malvern, UK) as follows: LP—d_10_ = 3.2 µm, d_50_ = 8.9 µm, d_90_ = 24.5 µm; MP—d_10_ = 2.9 µm, d_50_ = 9.3 µm, d_90_ = 26.8 µm; BP—d_10_ = 4.1 µm, d_50_ = 11.7 µm, d_90_ = 29.4 µm. The chemical compositions of MK, RM, LP, MP, and BP were determined using X-ray fluorescence (XRF, Axios mAX, PANalytical) Axios mAX (PANalytical B.V., Almelo, The Netherlands) and are presented in Table 1. Particle morphologies were observed by scanning electron microscopy (SEM, JSM-6510LV, JEOL) SEM (JSM-6510LV, JEOL Ltd., Tokyo, Japan). In addition to metakaolin and red mud, ground granulated blast-furnace slag (GGBFS) was incorporated at 10% by mass of the binder to accelerate the setting process and enhance early-age strength development. River sand (RS) was used as the fine aggregate in all mixes, with a maximum particle size of 2 mm and conforming to ASTM C109 [41] grading requirements. The material’s chemical composition is shown in Table 1.

### 2.2. Manufacturing of Geopolymer Mortar

The proposed mix of geopolymer-manufactured samples is illustrated in Table 2. The samples were prepared according to ISO 679:2009 [42], using the same methodology as that for preparing cement mortar. Ten different mixes were designed: a control sample with no waste filler material added and nine mixes with waste filler materials devised into three categories: LP, MP, and BP. Every category was also designed into three categories according to the ratio used to replace river sand for the manufactured sample. The ratio of the alkaline activators was fixed for all the geopolymer samples: sodium silicate and 12 Mol of sodium hydroxide prepared 24 h before use with a ratio of 2:1. In a mixer, firstly, MK was mixed with alkaline activators for 2 min; the red mud was added and mixed for 5 min; then the slag was mixed for 1 min; and, finally, the filler materials combined with river sand were added and mixed for 5 min. Moreover, the manufactured samples were pasted into cubic molds of 50 × 50 × 50 mm and prismatic molds of 40 × 40 × 160 mm and put in the vibration table to remove all the air pores and make the mortar dense and compacted. The samples were kept in the molds for 24 h at room temperature and cured in the oven for 6 h at 80 °C. After the specimens were taken out of the oven, they were kept at room temperature.

### 2.3. Acid Exposure Procedure (Immersion in H_2_SO_4_)

**Figure 1:**
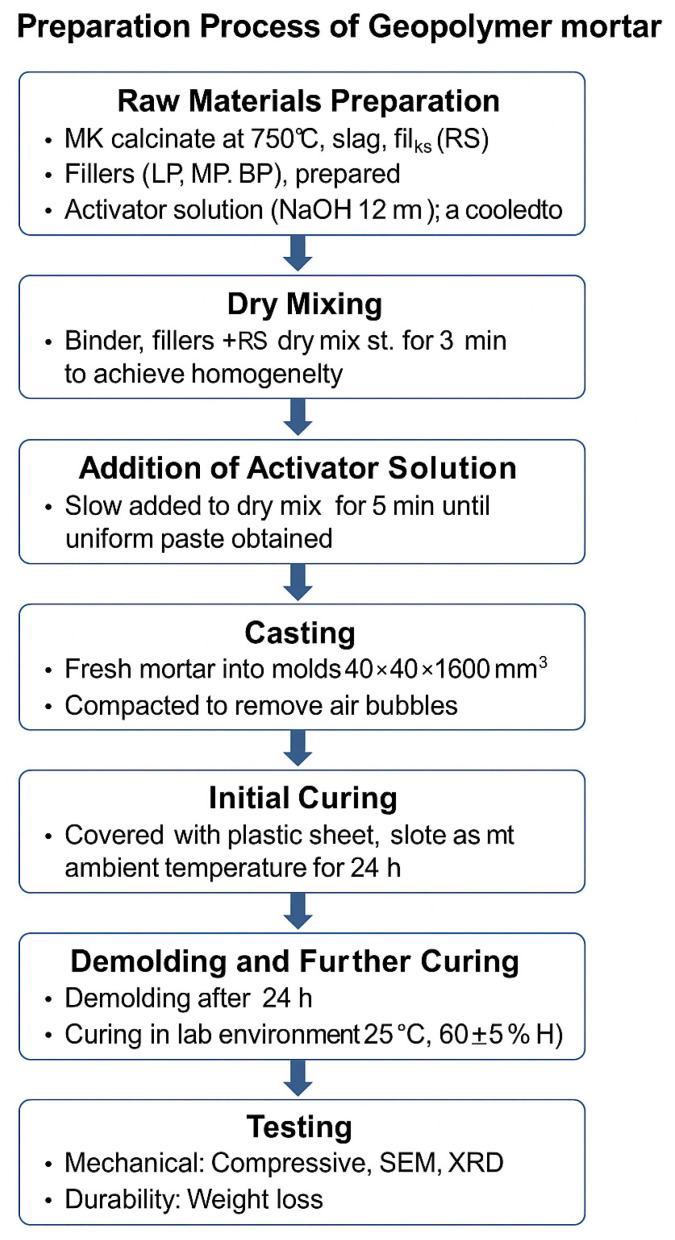
Schematic flowchart of the preparation process for geopolymer mortar specimens.

Cubic (50 × 50 × 50 mm) and prismatic (40 × 40 × 160 mm) specimens were immersed in 5% (*w*/*w*) H_2_SO_4_ prepared gravimetrically (add acid to water; 51 g conc. H_2_SO_4_, 95–98%, brought to 1.000 kg with deionized water). The initial solution pH was ~0.2 at 25 °C (first dissociation strong; partial second dissociation). A solution-to-specimen mass ratio ≥ 10:1 was used in covered HDPE containers held at 23 ± 2 °C without carbonation control. Because early leaching of alkalis from geopolymers partially neutralizes the acid, pH was monitored daily during the first 7 days and every 2–3 days thereafter. To maintain constant aggressiveness, the acid bath was fully replaced whenever pH exceeded 0.5 or at least twice per week during the first 2 weeks and weekly thereafter; we avoided drip additions to prevent local over-acidification and heat release. Gentle manual agitation was applied at each pH reading. Specimens were removed at 30, 60, and 90 days, briefly rinsed with deionized water, surface-dried, and tested for mass, UPV, compressive, and flexural strength; companion fragments were taken for SEM/XRD/FTIR (Figure 1). All mixes followed the same protocol to ensure comparability (bath logs with pH and replacement times are provided).

### 2.4. Evaluation Methods

#### 2.4.1. Mechanical Characteristics

The 10 mixes of geopolymer mortar samples were kept at room temperature before the mechanical characteristics evaluation. The compressive strength was evaluated according to ASTM C109 [41], and the flexural strength was assessed by ASTM C348 [43]. Thus, after 28 days and before exposure to sulfuric attack, the compressive strength was conducted for cubic samples to investigate the density and compactness of the geopolymer specimens, while the flexural strength three-point test was applied to the prismatic samples to evaluate the design details of the strength of the material to determine the properties of the material against bending and to establish homogeneity of samples. The stresses remain within the elasticity limit, and the section where the bending stress is calculated should be away from areas where stress concentration may occur. The Ultrasonic Pulse Velocity (UPV) is ascertained through a dependable, non-destructive evaluation of the ultrasonic wave pulse traversing the medium. The velocity signifies the formation of air forms or fissures. Consequently, an increased velocity signifies superior material quality. The decrease in UPV measured the damage resulting from high-temperature exposure.

Calculated with the following equation:

(1) $$D_{d}\;=\;1\;-\;(\frac{v_{a}}{v_{b}})$$

where:

$v_{b}$: UPV before exposure

$v_{a}$: UPV after exposure

D_d_: relative damage index

This method provides a non-destructive estimate of internal microcracking and structural deterioration due to thermal effects [44].

Furthermore, after exposure to sulfuric attack, compressive, flexural, and UPV tests were conducted to investigate each sample’s damage and evaluate the manufactured geopolymer samples according to the different filler materials used. The filler materials were investigated according to the three ratios used to replace river sand. Weight changes occur with mechanical tests, and the (%) changes were calculated before and after exposure to sulfuric acid, which is approximately increased over time.

#### 2.4.2. Microstructural Analysis (Microstructure Analysis of Mortar)

Representative fragments were cut from the central portion of 40 × 40 × 160 mm prisms (control and MP/LP/BP mixes); for post-acid observations, specimens previously immersed in 3% H_2_SO_4_ were rinsed with deionized water and dried before sampling. For SEM, pieces (~10 × 10 × 5 mm) were oven-dried at 50 °C to constant mass, optionally impregnated with low-viscosity epoxy, ground with SiC papers to 1200-grit, polished to ~1 µm, ultrasonically cleaned in ethanol, and sputter-coated with ~10 nm Au. Imaging was performed at Yıldız Technical University Central Laboratories (Istanbul, Turkey) in high vacuum at 15 kV (adjusted 10–20 kV as needed), working distance ~10 mm, using the SE detector (BSE selectively); for each mix/age, at least three non-overlapping fields of view were recorded at the magnifications reported in the figure captions. For XRD, material from the same specimens was gently crushed, dry-ground to pass 75 µm, and side-packed in low-background holders; measurements were made at Yıldız Technical University Central Laboratories on a Malvern PANalytical *Empyrean* diffractometer (θ–θ goniometer, radius 240 mm; reflection–transmission spinner on) using Cu Kα radiation (λ(Kα_1_) = 1.54060 Å, λ(Kα_2_) = 1.54443 Å) at 45 kV, 40 mA, fixed divergence slit 0.125°, 25 °C, continuous PSD scanning (length 3.35°) over 2θ = 2.01–89.99° with 0.013° 2θ steps and ~78.8 s/step; background subtraction and Kα_2_ stripping were applied, and phases were identified by search–match against the ICDD PDF database. FTIR spectra were collected in ATR mode (diamond) over 4000–400 cm^−1^ at 4 cm^−1^ resolution with 32 co-added scans per specimen; spectra were baseline-corrected and normalized for comparison, focusing on Si–O–T and sulfate-related bands.

#### 2.4.3. Statistical Treatment

All tests were performed on n = 3 specimens per mix and age unless stated otherwise. Results are reported as mean ± standard deviation with 95% confidence intervals where noted. Data normality and homogeneity of variance were checked (Shapiro–Wilk, Levene, IBM SPSS Statistics 25.0 software (IBM Corp., Armonk, NY, USA)). Group differences were assessed by one-way ANOVA with Tukey HSD (α = 0.05). Outliers were screened using Grubbs’ test; effect sizes are reported where appropriate.

## 3. Results and Discussion

### 3.1. Visual Inspection

**Figure 2:**
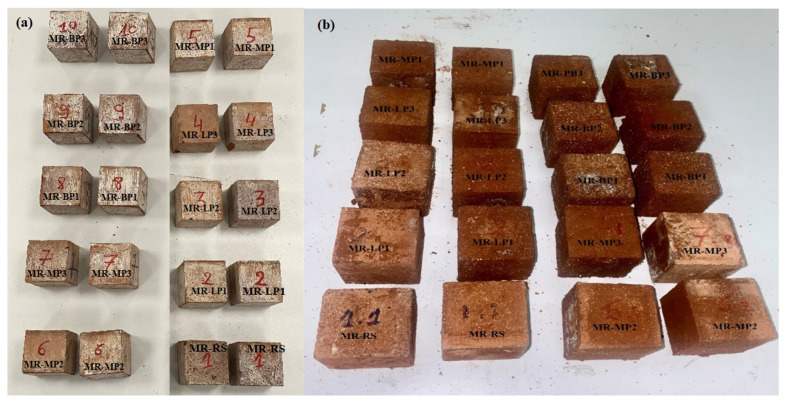
The manufactured geopolymer samples (**a**) before and (**b**) after exposure to acid attack.

Before and after being exposed to acid attack and at room temperature, the visual of manufactured geopolymer mortar samples is illustrated in Figure 2.

Figure 2 shows the visual changes before and after exposure to sulfuric acid for 30, 60, and 90 days. The resistance to the attack was influenced by the three different types of aggregate used in the manufacture of samples. The limestone powder samples display whitish or light-hued surface deposits, perhaps resulting from chemical interactions with sulfuric acid that produce sulfate salts or efflorescence. Numerous samples have a coarser, more degraded surface roughness than others, indicating material degradation from acid exposure. The control and limestone samples have more rounded or eroded edges, potentially signifying increased degradation. Conversely, accurately quantifying the image without X-ray diffraction and Fourier-transform infrared spectroscopy investigations is a complex process. The marble powder specimens exhibit more excellent uniformity than the limestone powder samples, which may have experienced dimensional loss or chipping. The arrangement indicates a methodical experiment: The basalt powder samples exhibit reduced damage compared to the control, limestone, and marble samples, indicating more significant degradation in the limestone and marble samples.

This study examined the performance of cement samples incorporating various mineral powders—marble powder (MR), limestone powder (LP), and basalt powder (BP)—after 90 days of exposure to sulfuric acid. Visual inspection indicated notable variations in degradation patterns across the compositions, underscoring the significance of mineral additives in enhancing acid resistance.

Samples with marble powder (MR-MP1, MP2, MP3) exhibited the most significant visual resistance to sulfuric acid. Surface textures were predominantly smooth, with only minimal roughness and edge wear noted. The absence of significant efflorescence or surface flaking indicates that the marble-based matrix likely possesses a denser structure, inhibiting acid penetration. Marble, primarily consisting of crystalline calcium carbonate, exhibits superior performance compared to limestone, likely due to its reduced porosity and enhanced microstructural stability in acidic environments [45].

The limestone powder samples (LP-LP1, LP2, LP3) exhibited moderate degradation. The samples exhibited a slight color change and displayed white salt deposits, likely due to the formation of gypsum (CaSO_4_) resulting from the reaction between sulfuric acid and calcium carbonate. The structure is generally sound; however, surface damage suggests that limestone is more reactive to acids, rendering it unsuitable for prolonged exposure to acidic conditions. The basalt powder samples (BP1, BP2, BP3) demonstrated the highest visual degradation. Severe surface roughness, flaking, and edge damage were observed, particularly in BP3. The degradation may arise from the heterogeneous mineral composition of basalt, which contains feldspars and mafic minerals that exhibit greater susceptibility to acid attack [45].

Furthermore, basalt’s increased porosity and intricate crystalline structure may have enabled greater acid penetration and subsequent chemical degradation. The visual characteristics of all samples exhibited a strong correlation with the type of mineral additive employed [45]. All mixtures demonstrated varying degrees of acid-induced degradation after 90 days. Marble powder (MR) exhibited the highest resistance to acid, while limestone powder (LP) displayed intermediate performance, characterized by signs of efflorescence. In contrast, basalt powder (BP) showed the lowest resistance, with observable structural weakening.

The findings demonstrate that the mineral additive type significantly influences the chemical resistance of geopolymer mortar composed of metakaolin and red mud, mainly when LP, MR, and BP are utilized as fillers in acidic conditions. Marble powder exhibits superior performance, potentially rendering it a more effective additive in sewer systems, industrial flooring, or acidic soil environments where acid resistance is essential.

### 3.2. Mechanical Tests

#### 3.2.1. Compressive and Flexural Strength

**Figure 3:**
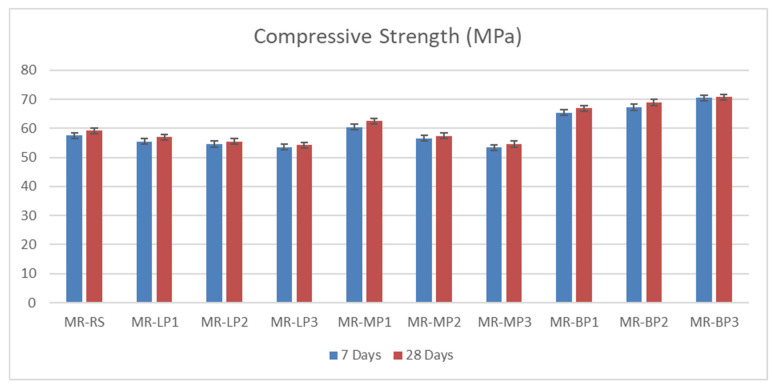
The compressive strength of manufactured geopolymer samples before exposure to acid attack.

**Figure 4:**
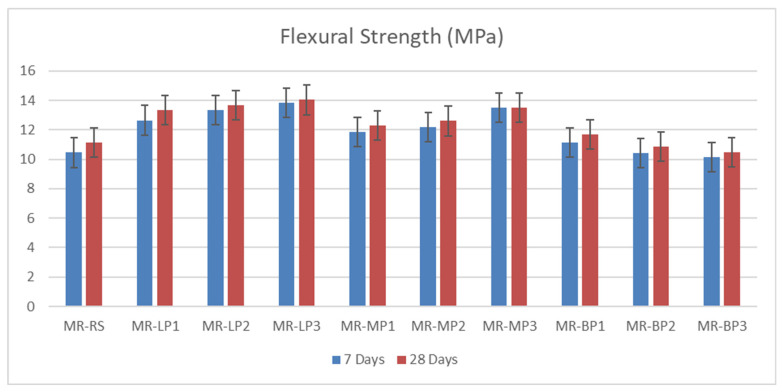
The flexural strength of manufactured geopolymer samples before exposure to acid attack.

The compressive and flexural strength of the manufactured geopolymer mortars were investigated at 7 and 28 days before and after exposure to sulfuric attack. The results obtained were shown in Figure 3. Due to its high hardness and chemical structure, Basalt powder performs better mainly in applications requiring compressive strength. Basalt powder provides strong bonding and hardness thanks to its high silica and alumina content. It is resistant to pressure due to its complex, durable minerals. Since marble powder has a more crystalline and compact structure than limestone, it is slightly superior to limestone in terms of compressive strength. It contains calcium carbonate and provides suitable microstructure and filling, but its hardness is not as high as basalt. Limestone powder, on the other hand, can be more porous, which can reduce compressive strength slightly. It contains calcium carbonate and may perform slightly lower than marble powder mechanically; however, it offers reasonable compressive strength with good filling properties. It has been shown that basalt-added concrete and geopolymers increase compressive strength by 10–20%. Many studies have also stated that marble powder increases compressive strength due to its filling and microstructure-improving effect [45]. The obtained results of flexural strength were shown in Figure 4; Basalt powder contains silicate and mafic minerals (olivine, pyroxene). These minerals have low reactivity in alkaline environments. In other words, they do not form a strong chemical bond with the binding matrix. In addition, the heterogeneous crystalline structure of basalt can cause cracks to propagate quickly. This means lower flexural strength. Limestone powder contains a high percentage of calcium carbonate (CaCO_3_). This compound can react with binders, especially metakaolin or red mud, and support the formation of calcium aluminate hydrates (CAH) and calcium silicate hydrates (CSH). These reaction products provide better microstructural integration in the binding matrix, thus delaying crack propagation and increasing flexural strength. Limestone powder provides an advantage over basalt powder in flexural strength by offering better chemical compatibility with the binder, better void-filling ability, and lower porosity. Basalt, on the other hand, is less reactive and may exhibit more brittle behavior. Marble powder has a crystalline structure dominated by calcium carbonate (CaCO_3_) and generally has finer and more homogeneous particle sizes. In this way, it better fills the microvoids in the concrete or geopolymer matrix and forms a tight structure. As a result, crack propagation becomes more complex, and bending strength increases. Basalt powder contains silica, alumina, and mafic minerals. These minerals form weaker chemical bonds with the matrix, and the pore structure of basalt powder generally has higher porosity than marble. This leads to easier propagation of microcracks during bending [34].

The manufactured geopolymer samples were immersed in a 5% sulfuric acid solution for 30, 60, and 90 days to evaluate their long-term acid resistance and durability under aggressive chemical conditions. At each interval, the samples were evaluated for compressive and flexural strength retention.

**Figure 5:**
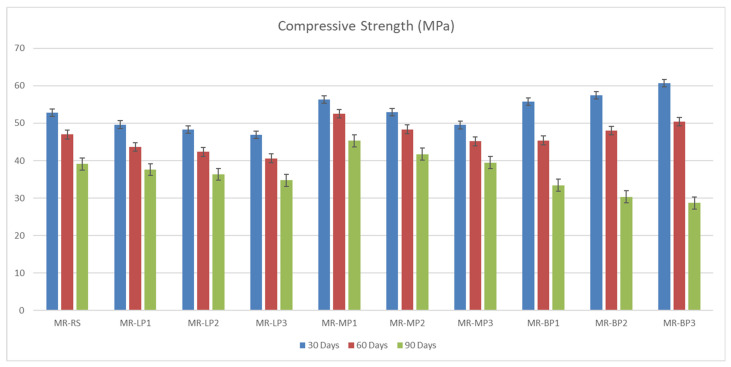
The compressive strength of manufactured geopolymer samples after exposure to acid attack.

The results obtained were shown in Figure 5. Rapid CaCO_3_ dissolution with subsequent gypsum formation produces an initial pore-blocking effect (higher strength retention for LP/MP) but leads to progressive softening and fissure growth over time, explaining the 30→90 d retention differences (MP3 ≈ 80%, LP3 ≈ 74%, BP3 ≈ 47%).

The specimens containing marble powder were predominantly constituted of calcium carbonate (CaCO_3_), although in a more crystalline and denser form than limestone. Particle Granularity: Marble powder is frequently finely milled, resulting in a denser and less porous structure. While CaCO_3_ interacts with sulfuric acid to produce gypsum, the dense crystalline structure of marble inhibits acid infiltration [46]. The tiny marble particles may enhance packing density, decrease porosity, and restrict acid intrusion. Marble powder can function as a micro-filler, optimizing the pore structure and improving durability—reduced interior damage from an acid assault results in improved retention of compressive strength over time [47]. Limestone powder mainly consists of CaCO_3_; nonetheless, it is often softer, more porous, and less crystalline than marble, and it interacts with sulfuric acid to produce calcium sulfate (gypsum) and CO_2_, potentially resulting in expansion, cracking, and a reduction in strength. In moderate amounts, limestone powder enhances workability and fills cavities in the matrix, offering some resistance [48]. It provides filler advantages but exhibits more acid reactivity than marble, resulting in mediocre durability. Conversely, basalt is a volcanic rock abundant in silica (SiO_2_), alumina (Al_2_O_3_), iron oxides, and other metal oxides, with minimal to no CaCO_3_. It is more chemically robust in acidic environments, although it exhibits little reactivity within the geopolymer matrix, indicating it does not actively engage in geopolymerization [49]. It functions primarily as an inert filler, which may result in diminished bonding strength and increased porosity. This may lead to a compromised structure over time due to acid exposure, resulting in inadequate matrix integration and diminished calcium-based repair processes, such as gypsum production.

**Figure 6:**
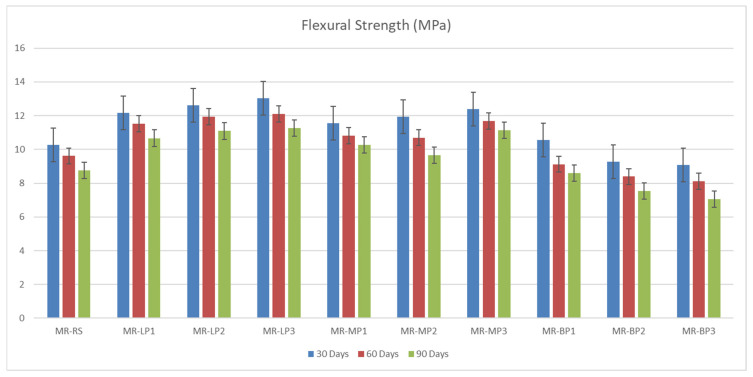
The flexural strength of manufactured geopolymer samples after exposure to acid attack.

The flexural strength (Figure 6) of geopolymer mortar is contingent upon the kind of included powder, with marble powder typically yielding superior performance, followed by limestone and then basalt powder. Marble powder, due to its small particle size and dense crystalline structure, enhances particle packing and strengthens the interfacial bonds within the geopolymer matrix. This enhances resistance to fracture propagation, leading to increased flexural strength, even in the presence of sulfuric acid, where gypsum production may temporarily occlude pores and diminish future acid penetration. Limestone powder enhances the microstructure and initial strength; however, it exhibits increased reactivity with sulfuric acid, resulting in gypsum production, internal stress, and a decline in flexural performance over time. Conversely, basalt powder, although chemically stable, exhibits the lowest flexural strength due to its inert characteristics and reduced adhesion to the geopolymer matrix, which can potentially result in microcracks and a less cohesive structure. The disparities highlight the significant impact of filler characteristics on the flexural durability of geopolymer mortars under harsh conditions.

#### 3.2.2. Ultrasonic Pulse Velocity (UPV)

The Ultrasonic pulse velocity (UPV) of the manufactured geopolymer mortars was investigated 28 days before and after exposure to sulfuric attack. The results obtained are shown in Figure 6. At 28 days, the UPV values are affected by density, porosity, and the elastic properties of concrete. Elevated UPV levels often indicate superior quality and augmented strength of concrete [50]. Mortar with basalt powder demonstrates increased UPV values attributed to the dense and hard characteristics of basalt, which improve the mortar’s overall density and elastic modulus. Marble powder, primarily calcium carbonate, yields a denser matrix than limestone powder, leading to moderate to high UPV values. Mortar-containing limestone powder typically exhibits marginally reduced UPV values compared to those with basalt and marble powders, which can be ascribed to its comparatively greater porosity and lower density [51].

The UPV increases as compressive strength increases. This is commonly represented through a regression model, typically as follows:

F_c_ = a × v^b^ (2)

where:

F_c_: Compressive strength (MPa)

V: UPV (km/s)

a, b: regression constants depending on mix type and material properties

**Figure 7:**
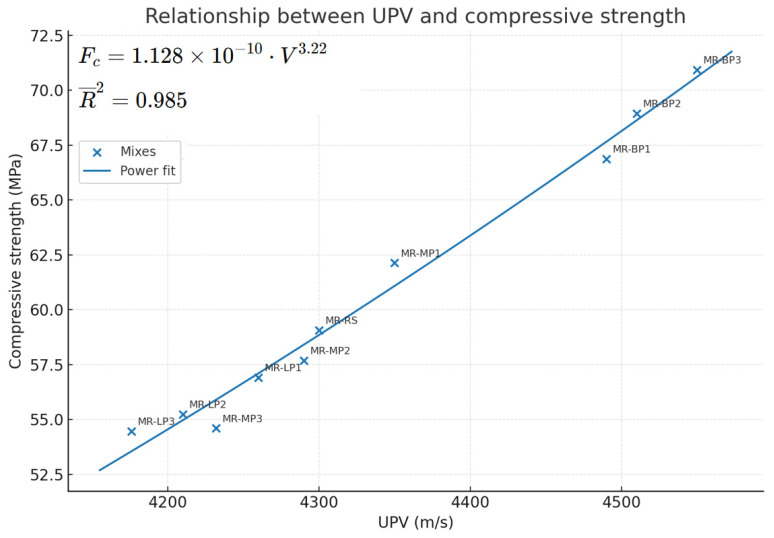
Relationship Between UPV And Compressive Strength.

Concrete incorporating basalt powder frequently aligns closely with the curve or is positioned marginally above it. Due to its stiffness and density, it leads to the strongest correlation. Marble powder concrete approximates the curve or is marginally below it and also offers a good balance between workability and strength. Limestone powder concrete may exhibit reduced performance due to increased air voids or diminished matrix stiffness. It generally shows the lowest UPV and strength but is still suitable for non-structural or blended use. As shown in Figure 7, UPV increased proportionally with compressive strength, particularly in basalt-filled samples. Basalt-filled mortars (MR-BP3) showed the strongest correlation (R^2^ = 0.98), indicating a more homogeneous and dense matrix. Marble powder samples (MR-MP1) had a somewhat lower correlation (R^2^ = 0.92) but remained within a reliable prediction range. Limestone samples (MR-LP1) showed the most minor correlation (R^2^ = 0.89), possibly due to its increased porosity and microstructural variability. The regression trends indicated that higher UPV is consistently related to greater compressive strength, regardless of filler type.

**Figure 8:**
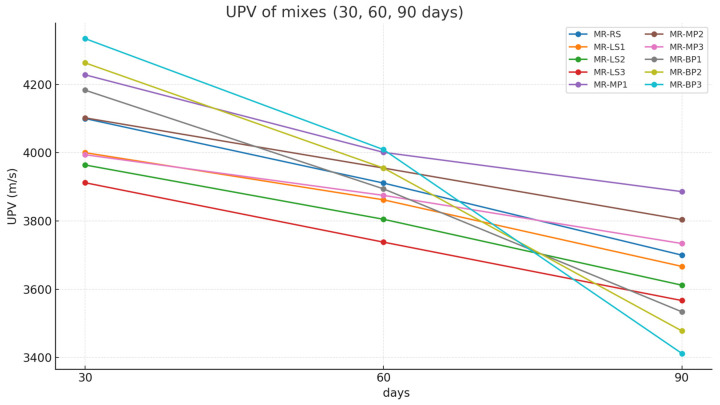
Ultrasonic pulse velocity (UPV) of all mixes measured at 30, 60, and 90 days.

The (UPV) of the manufactured geopolymer mortars after exposure to sulfuric acid was investigated at 30 days, 60 days, and 90 days. The results obtained are shown in Figure 8. Ultrasonic Pulse Velocity (UPV) serves as a non-destructive technique for evaluating the internal quality and integrity of geopolymer mortars by quantifying the propagation speed of ultrasonic waves within the material. This study demonstrates that mortars incorporating marble powder achieved the highest UPV values, attributed to enhanced particle packing and decreased porosity, leading to a denser and more uniform matrix that promotes accelerated wave transmission. Limestone powder initially improved the microstructure, resulting in moderate UPV values; however, its increased reactivity with sulfuric acid led to more significant degradation over time, subsequently reducing UPV readings [52]. Geopolymer mortars incorporating basalt powder exhibited the lowest UPV values, attributed to inadequate binder interaction and reduced matrix density, notwithstanding basalt’s chemical stability in acidic conditions. The results are consistent with the mechanical performance, indicating that UPV accurately represents the internal condition and durability of the mortars following exposure to acidic environments.

#### 3.2.3. Weight Change

**Figure 9:**
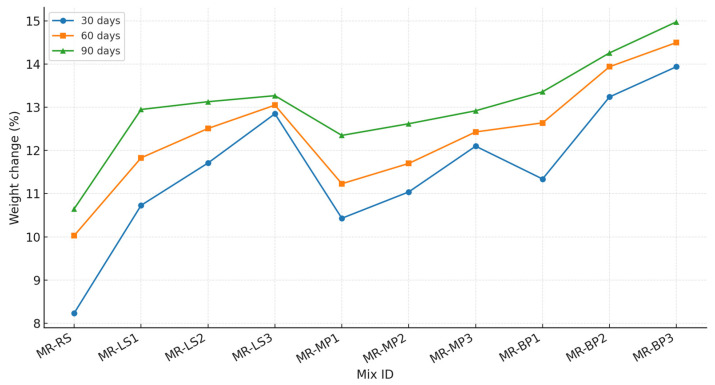
Weight changes (%) of the samples after 30, 60, and 90 days of exposure to sulfuric acid.

After 30, 60, and 90 days of exposure to sulfuric acid, the performance of geopolymer mortar samples showed significant variation depending on the type of filler used. The results obtained are shown in Figure 9. The group with marble powder demonstrated superior durability, characterized by minimal weight loss and optimal retention of mechanical properties over time. The dense packing and filler effect of marble were responsible for limiting acid ingress and minimizing chemical degradation. The limestone powder group exhibited moderate resistance. Initially, it performed well; however, progressive acid attack resulted in notable gypsum formation and leaching, especially after 60 and 90 days, leading to increased weight loss and surface deterioration. The geopolymer mortars containing basalt powder exhibited the least favorable performance, characterized by the most significant weight loss and most pronounced degradation throughout all exposure durations. The low reactivity of basalt and its limited role in matrix densification rendered these samples more vulnerable to acid infiltration and leaching, resulting in accelerated deterioration, particularly evident after 90 days. The observed trends suggest that the type of filler has a significant influence on the long-term acid resistance of geopolymer mortars.

The reduction in mass of geopolymer mortars subjected to sulfuric acid serves as a significant measure of material deterioration, indicating the degree of chemical assault and matrix dissolution. This study found that mortars containing marble powder demonstrated the least weight loss, attributed to the dense structure and filler properties of marble, which restricted acid penetration and gypsum-related deterioration. Limestone powder, primarily consisting of calcium carbonate, exhibited moderate weight loss due to its increased porosity and reactivity with sulfuric acid, resulting in the formation of gypsum and spalling. In contrast, mortars with basalt powder exhibited the most significant weight loss due to basalt’s low reactivity in geopolymer systems, leading to a weaker, less compact matrix that was more susceptible to acid penetration and leaching. The findings indicate that marble powder improves acid resistance through increased matrix density, while basalt’s inert properties provide minimal protection against acid-induced mass loss.

### 3.3. Microstructural Analysis

Microstructural analysis was conducted using Scanning Electron Microscopy (SEM), X-ray Diffraction (XRD), and Fourier Transform Infrared Spectroscopy (FTIR) to evaluate the morphological and phase changes in geopolymer mortar samples containing marble, limestone, and basalt powders after exposure to sulfuric acid for 90 days.

#### 3.3.1. Scanning Electron Microscope (SEM) Analysis

**Figure 10:**
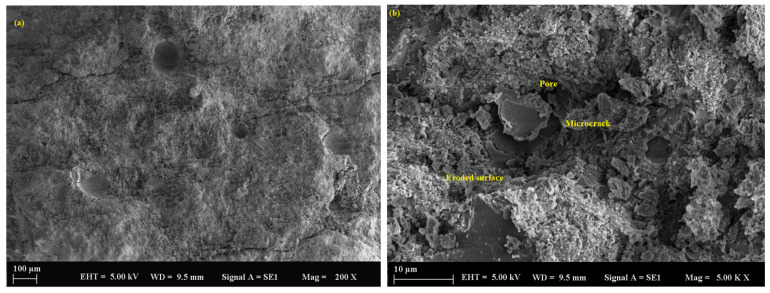
SEM micrograph of the control sample: (**a**) 100 µm and (**b**) 10 µm.

**Figure 11:**
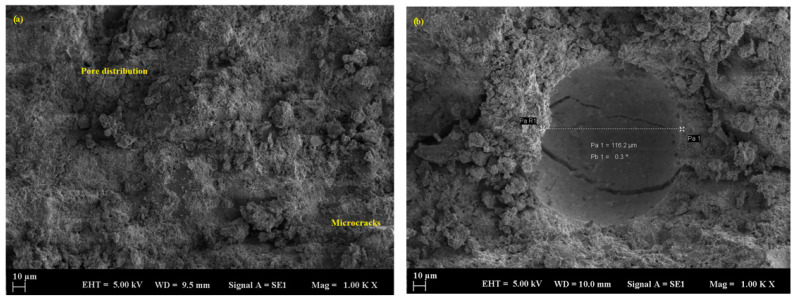
SEM micrograph of the control sample: (**a**) 10 µm and (**b**) 10 µm.

The control sample, containing only river sand as filler, initially exhibited a relatively dense microstructure. However, significant deterioration was observed over time following exposure to sulfuric acid. SEM analysis (Figure 10a,b) revealed the progression of microcracking, surface erosion, and a loss of matrix integrity, particularly after 90 days. This degradation was attributed to the larger particle size and lower packing density of river sand compared to fine mineral powders, which allowed for deeper acid penetration. The average pore size in the control sample was measured at 116.2 µm (Figure 11a,b), further contributing to its vulnerability. Additionally, the interfacial transition zones between the river sand and the geopolymer matrix showed reduced uniformity and increased susceptibility to chemical attack.

**Figure 12:**
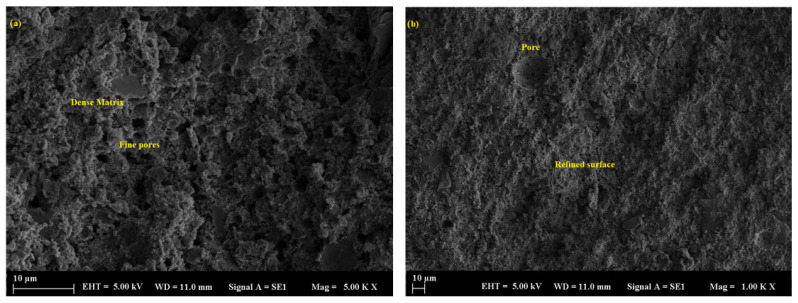
SEM micrograph of the MP sample: (**a**) 10 µm and (**b**) 10 µm.

**Figure 13:**
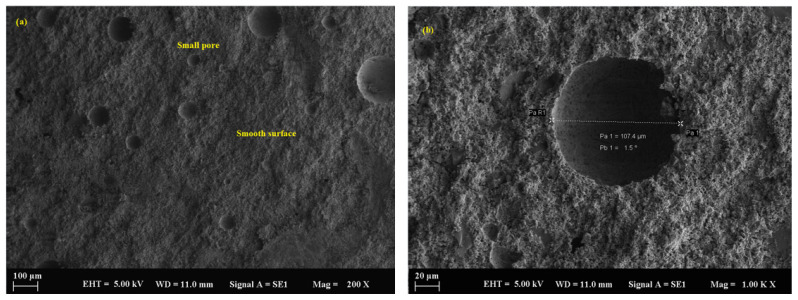
SEM micrograph of the MP sample: (**a**) 100 µm and (**b**) 20 µm.

According to mechanical results, geopolymer mortars with marble powder replacement exhibited a compact and refined microstructure characterized by closely packed particles and minimally visible pores. The enhanced packing effect restricted acid ingress and safeguarded the binder matrix. At a 50% replacement level, marble-filled samples maintained significant structural integrity; however, minor surface etching and localized microcracking were noted after 90 days (Figure 12a,b). SEM images revealed that samples containing marble powder exhibited a denser and more cohesive matrix characterized by minimal surface cracking and restricted deposition of reaction products, such as gypsum. The average pore size in these samples was significantly reduced compared to the control, typically measuring below 107 µm (Figure 13a,b), which further contributed to the reduced permeability and improved acid resistance.

**Figure 14:**
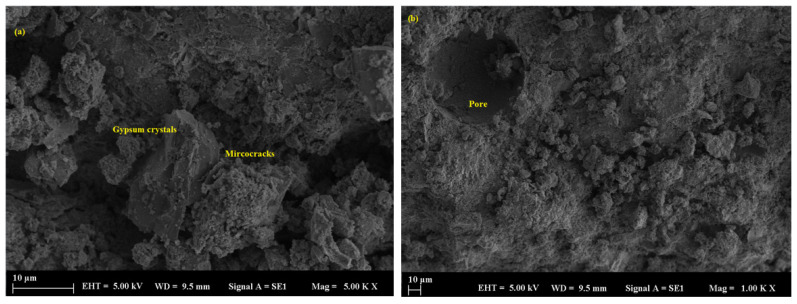
SEM micrograph of the LP sample: (**a**) 10 µm and (**b**) 10 µm.

**Figure 15:**
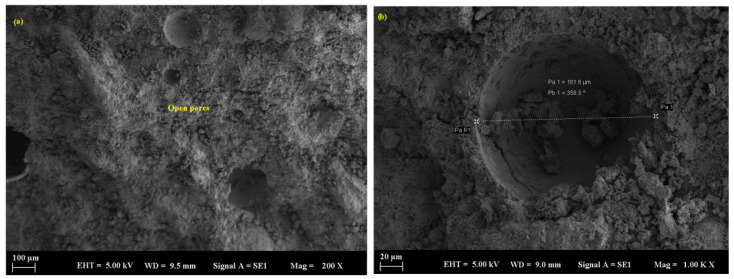
SEM micrograph of the LP sample: (**a**) 100 µm and (**b**) 20 µm.

Limestone-containing mortars exhibited more significant microcracks and observable gypsum crystal formation, particularly after 90 days, suggesting a progressive acid attack (Figure 14a,b). Limestone powder-filled mortars showed more pronounced degradation as the replacement level increased. The microstructure exhibited an increased number of pores, gypsum precipitation along fissures, and signs of leaching, particularly following prolonged exposure to acid (Figure 15a,b). The average pore size in these samples was measured at 161.6 µm, which facilitated deeper acid penetration and accelerated chemical deterioration. The higher calcium content of limestone reacts with sulfuric acid to produce gypsum (CaSO_4_·2H_2_O), resulting in surface softening and material spalling.

**Figure 16:**
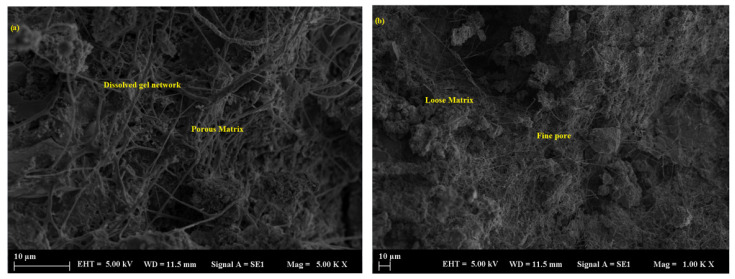
SEM micrograph of the BP sample: (**a**) 10 µm and (**b**) 10 µm.

**Figure 17:**
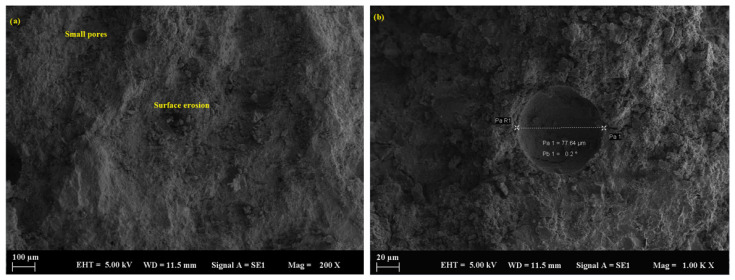
SEM micrograph of the BP sample: (**a**) 100 µm and (**b**) 20 µm.

Samples containing basalt demonstrated the most degraded microstructure, marked by heightened porosity, surface erosion, and unreacted particles within a poorly bonded matrix, especially following extended exposure. Mortars filled with basalt powder, particularly at a 50% replacement level, exhibited the highest porosity and weakest bonding characteristics. Scanning electron microscopy (SEM) indicated the presence of unreacted basalt particles distributed within a loosely arranged matrix, exhibiting observable signs of disintegration (Figure 16a,b). Interestingly, despite the overall deterioration, the average pore size in basalt-filled samples was relatively small—typically below 77.64 µm—owing to the finer particle size and better packing efficiency of the basalt powder. However, the limited reactivity of basalt resulted in minimal contribution to the densification of the mortar structure, thereby increasing its susceptibility to acid attack (Figure 17a,b). Acid exposure intensified the dissolution of the geopolymer gel and the leaching of alkalis, resulting in diminished interfacial bonding and surface erosion, which was particularly noticeable at elevated replacement levels.

**Figure 18:**
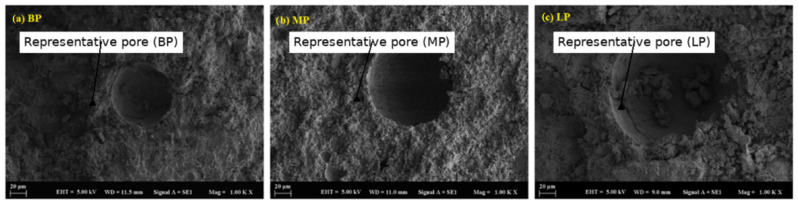
SEM micrographs showing pore structures of (**a**) basalt powder (BP), (**b**) marble powder (MP), and (**c**) limestone powder (LP) samples at 1.00 KX magnification.

Marble and limestone, despite having larger pores, resist acid better because they react chemically to form protective products. Limestone and marble are primarily calcium carbonate (CaCO_3_). When sulfuric acid reacts, it combines with calcium carbonate to form calcium sulfate (gypsum), carbon dioxide, and water. While this reaction appears to be degradation, the gypsum can sometimes fill pores and microcracks, acting as a protective barrier in the early stages of acid attack. These materials buffer pH and delay deep acid penetration more effectively than silica-rich fillers, such as basalt, which are more chemically inert but do not neutralize acid. Despite this, basalt creates a physically strong but chemically vulnerable matrix in acidic environments. Basalt powder particles are typically finer and have a more angular shape, which enables them to fill voids more efficiently. This leads to better packing density, resulting in a denser matrix with smaller pores. Basalt also contains more aluminosilicate content, which helps create a stronger geopolymer gel network. In terms of pore size, basalt-filled mortars exhibited the smallest pores (77.64 µm), followed by marble-filled mortars (107 µm), while limestone-filled mortars showed the largest pores (161.6 µm), correlating with their respective particle sizes and packing densities (Figure 18a–c).

#### 3.3.2. X-Ray Diffraction (XRD) and FTIR Analysis

**Figure 19:**
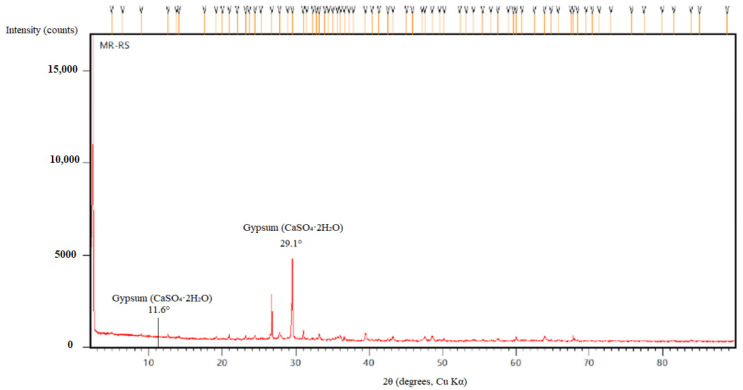
XRD patterns of MR-RS.

**Figure 20:**
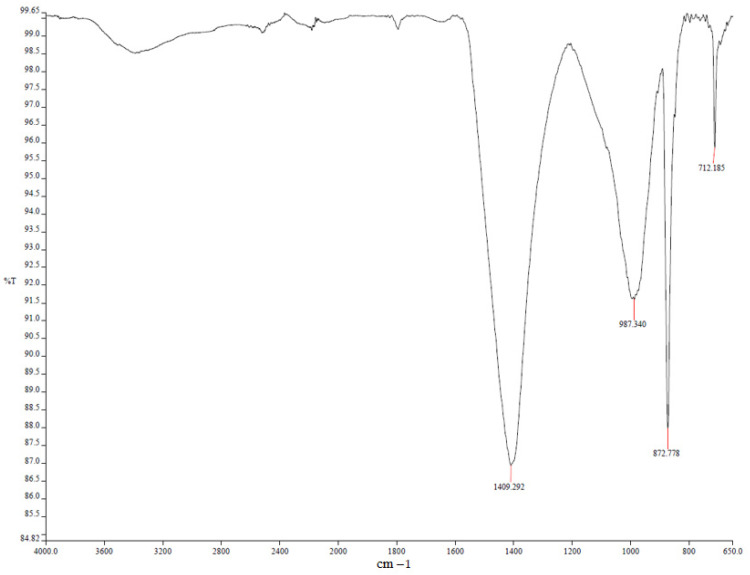
FTIR spectra of MR-RS.

In comparison to the modified mortars, the river sand-only sample exhibited increased porosity and more pronounced deterioration after prolonged exposure to sulfuric acid. While gypsum formation was observed, it was less extensive than in limestone-filled mixtures due to the lower calcium content of river sand. XRD analysis confirmed the modest formation of secondary crystalline phases, with weak diffraction peaks observed at 2θ ≈ 11.6° and 29.1°, corresponding to gypsum (CaSO_4_·2H_2_O) (Figure 19). FTIR spectra (Figure 20) revealed a weakening of the Si–O–T (T = Si or Al) stretching vibrations, with a noticeable shift from 990 cm^−1^ to 970 cm^−1^, and the appearance of new sulfate bands at approximately 1120 cm^−1^, indicating progressive structural degradation and the incorporation of sulfate species into the matrix.

**Figure 21:**
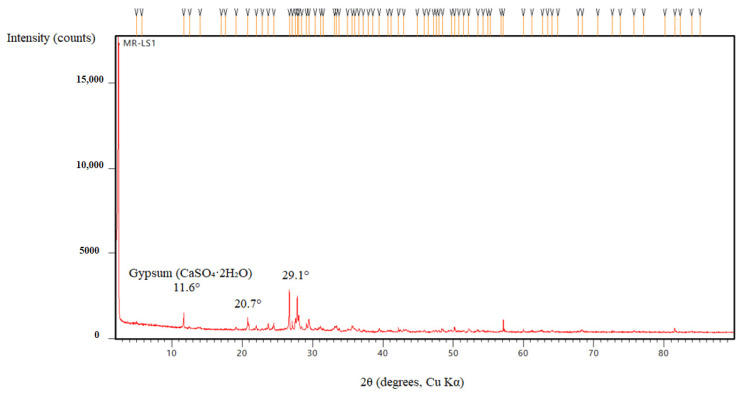
XRD patterns of LP.

**Figure 22:**
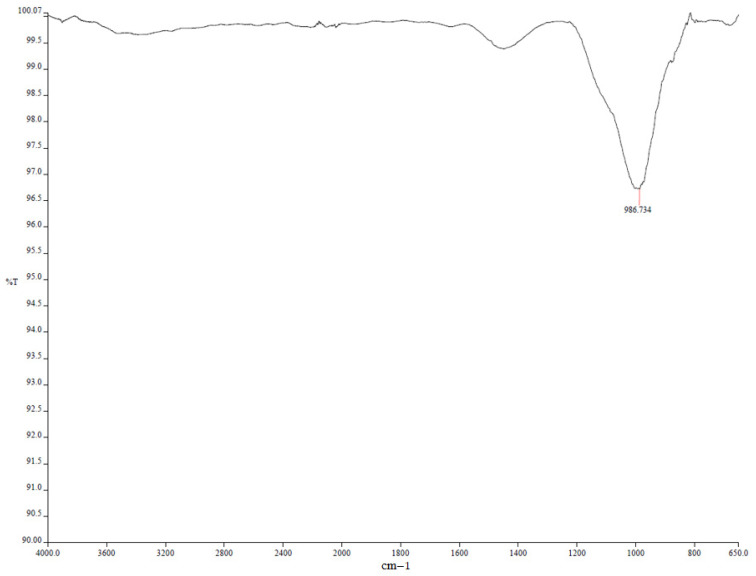
FTIR spectra of LP.

Limestone-containing geopolymer mortars exhibited more significant microcracking, surface deterioration, and gypsum crystal formation, particularly after 90 days of sulfuric acid exposure. The extent of degradation intensified with elevated levels of limestone powder replacement due to the substantial calcium carbonate component in the limestone powder. XRD analysis (Figure 21) revealed distinct crystalline gypsum peaks at 2θ ≈ values of approximately 11.6°, 20.7°, and 29.1°, confirming substantial sulfate reaction and secondary phase formation. FTIR spectra (Figure 22) supported these findings, showing a shift in the main Si–O–T stretching band from 990 cm^−1^ to 960 cm^−1^, along with intensified sulfate absorption bands near 1120 cm^−1^ and emerging carbonate bands around 875 cm^−1^, indicating both sulfate incorporation and partial carbonate retention. The microstructure exhibited an increased number of pores and gypsum precipitation along microcracks, accompanied by visible signs of leaching.

**Figure 23:**
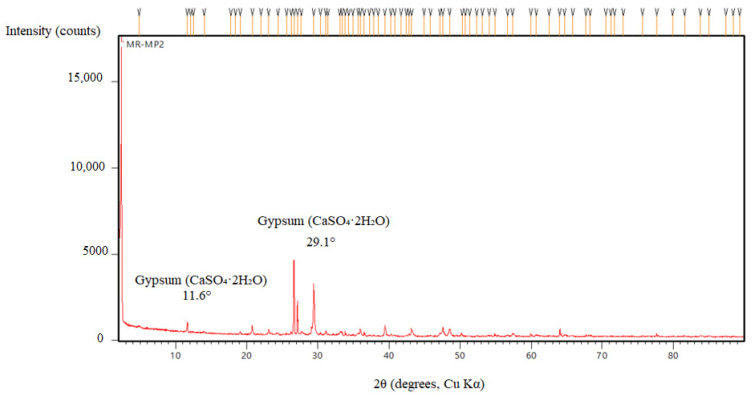
XRD patterns of MP.

**Figure 24:**
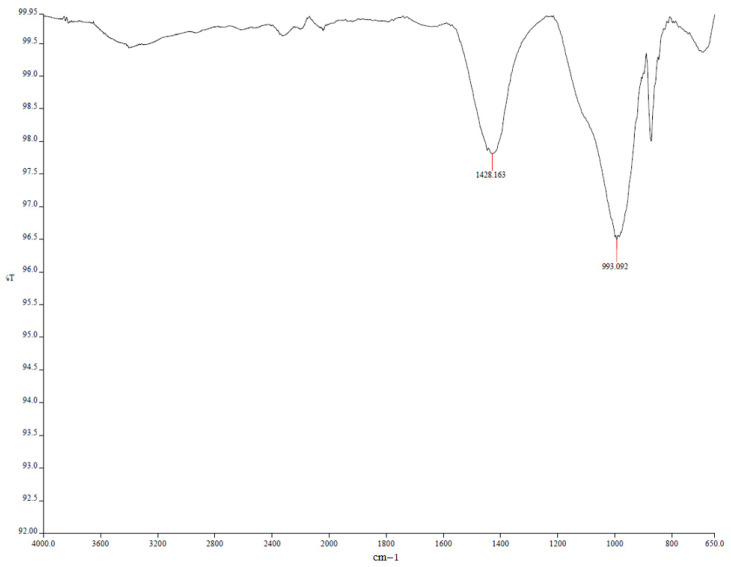
FTIR spectra of MP.

Marble powder-filled geopolymer mortars exhibited a dense and refined microstructure characterized by tightly packed particles and minimally visible pores, which contributed to improved resistance against sulfuric acid attacks. After 90 days of exposure, samples with up to 50% marble replacement retained most of their structural integrity, though minor surface etching and localized microcracking were observed. XRD analysis (Figure 23) revealed moderate-intensity gypsum peaks at 2θ ≈ 11.6° and 29.1°, indicating limited sulfate reaction and secondary phase formation. FTIR spectra (Figure 24) revealed a slight shift in the Si–O–T stretching vibration from 990 cm^−1^ to 975 cm^−1^, accompanied by the appearance of sulfate absorption bands near 1120 cm^−1^, indicating minor chemical degradation. The resistance of marble-containing mortars is attributed to the calcium carbonate buffering effect and the formation of gypsum, which partially fills pores, thereby limiting acid ingress.

**Figure 25:**
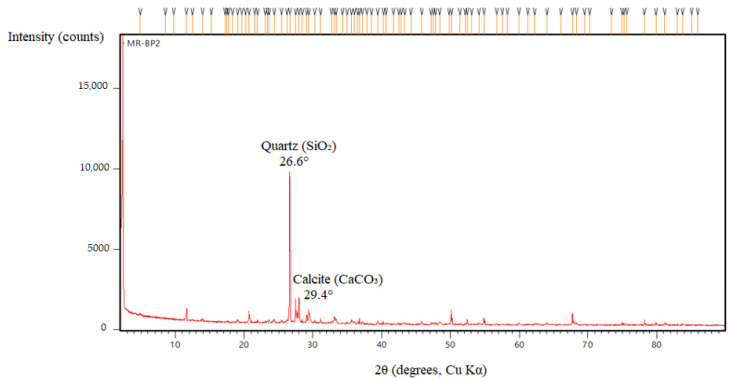
XRD patterns of BP.

**Figure 26:**
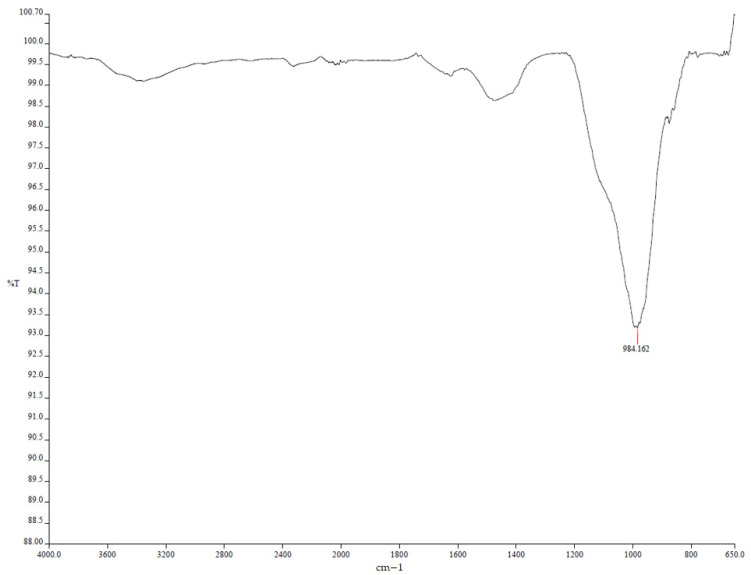
FTIR spectra of BP.

Mortars containing basalt powder exhibited the most degraded microstructure among the studied samples, characterized by increased porosity, surface erosion, and numerous unreacted particles within a loosely bonded matrix, especially after prolonged exposure to sulfuric acid. The limited chemical reactivity of basalt powder resulted in minimal densification of the geopolymer matrix, thereby increasing its susceptibility to acid attack. XRD analysis (Figure 25) revealed only weak and broad peaks, with no significant formation of secondary sulfate phases, such as gypsum, indicating limited chemical interaction between the basalt and sulfuric acid. FTIR spectra (Figure 26) revealed a reduction in Si–O–T band intensity at around 990 cm^−1^, accompanied by slight broadening, but without the pronounced sulfate absorption bands typically observed in calcium-rich samples, indicating structural degradation without substantial sulfate incorporation. These results were obtained using a PANalytical XRD instrument PANalytical X-ray diffraction (XRD) instrument (Malvern Panalytical B.V., Almelo, The Netherlands) with a copper anode (Kα_1_ = 1.54060 Å, Kα_2_ = 1.54443 Å, Kβ = 1.39225 Å) and a 0.02 mm nickel filter. The scan was conducted over a 2θ range of 2.01° to 89.99° with a derived step size of 0.013°. Peak identification utilized the minimum second derivative method, requiring a minimum significance of 0.5.

Due to their denser microstructure and lower calcium leachability, the results indicate that marble-based geopolymer mortars exhibit better acid resistance. On the other hand, the basalt group suffered the most significant microstructural damage over time, whereas the limestone group exhibited mild deterioration. Overall, due to the packing effect and chemical stability of the filler, increasing the amount of marble powder used in place of river sand improved the microstructural integrity and acid resistance. Because of its reactive calcium component, limestone powder made people more susceptible to sulfuric acid, particularly when used as a 50% replacement. Despite being chemically inert, basalt powder failed to adequately densify the mortar structure, resulting in increased porosity and acid-induced degradation at higher replacement levels. Overall, the river sand control sample lacked the microstructural densification benefits provided by marble powder, even if it occasionally performed better than high-percentage basalt or limestone mortars. This demonstrates how using reactive or dense mineral powders in partial substitution might improve resistance to acid assault.

We strengthened the evidence–conclusion link by adding quantitative comparisons and explicit figure references. Basalt mixes showed the largest strength loss during acid exposure: BP3 fell from 60.6 MPa (30 d) to 28.7 MPa (90 d), a −52.6% change, versus MP3: −20.2% and LP3: −25.8% (Figure 5). SEM of BP (Figure 16 and Figure 17) shows unreacted basalt particles and a porous, weakly bonded matrix, consistent with chemical attack. XRD (Figure 19, Figure 20, Figure 21, Figure 22, Figure 23, Figure 24 and Figure 25) indicates little/no gypsum formation in BP mixes, whereas LP/MP exhibit gypsum peaks (≈11.6°, 20.7°, 29.1°), which can partially block pores. FTIR (Figure 25) shows attenuation/broadening of the Si–O–T band in BP after acid exposure, consistent with gel depolymerization and alkali leaching. Together, these microstructural and chemical signatures explain the lower strength retention of BP (47–60%) compared to MP (≈79–81%) and LP (≈74–76%).

## 4. Conclusions

Mechanical performance under acid attack: After 90 days in 3% H_2_SO_4_, the control retained 61% of its 28-day compressive strength, while basalt-filled mortars (15 wt.%) retained 84% (46.8 MPa). Flexural strength followed a similar trend, with basalt mixes retaining 80% vs. 58% for the control.

Mass loss: Acid exposure caused a 6.4% mass loss in the control after 90 days, which was reduced to 3.2% with basalt filler and 3.8% with marble filler.

Ultrasonic pulse velocity (UPV): UPV dropped by 35% less in basalt-filled mortars compared to the control, indicating better microstructural integrity after acid exposure.

Microstructural observations: SEM images showed fewer and narrower cracks in mixes with basalt and marble powders; XRD and FTIR confirmed the persistence of N-A-S-H gel and reduced gypsum formation in these mixes.

Overall implication: Incorporating industrial by-product fillers, particularly basalt powder at 15 wt.%, enhances acid resistance and mechanical stability, providing a sustainable, durable alternative to traditional cementitious systems in aggressive environments.

## Figures and Tables

**Table 1:** Chemical composition of materials used in this study (wt %) [30].

Binders	CaO	SiO_2_	Al_2_O_3_	Fe_2_O_3_	MgO	K_2_O	Na_2_O	P_2_O_5_	MnO
Metakaolin	0.9	56.1	40.23	0.85	0.16	0.51	0.24	0.07	–
Red-Mud	3.22	17.38	24.52	35.25	0.42	0.43	8.45	–	0.07
Slag	35.58	40.55	10.11	12.83	5.87	–	0.79	0.08	1.14
Activators (%)	NaOH	Na_2_CO_3_	CL	SO_4_	Al	Fe	SiO_2_	Na_2_O	Density (g/mL)
Sodium Silicate	99.1	0.3	≤0.01	≤0.01	≤0.002	≤0.002	–	–	–
Sodium Hydroxide	–	–	–	–	–	≤0.005	27.01	8.02	1360
Fillers	SiO_2_	Al_2_O_3_	Fe_2_O_3_	TiO_2_	CaO	CaO_2_	K_2_O	Na_2_O	SO_3_
Limestone powder	3.03	0.82	0.58	–	–	92.9	–	–	1.18
Marble powder	1.12	0.73	0.05	–	–	83.22	–	–	0.56
Basalt powder	56.9	17.06	8.01	0.9	7	–	1.09	3.08	–
River Sand (RS)	96.0	2.04	1.04		0.4				

**Table 2:** The manufactured geopolymer mortars.

Mix ID	Red-Mud	Metakaolin	Slag (10%)	SS:SH	RS	LP	BP	MP
MR-RS	45%	45%	10%	1:2	100%	–	–	–
MR-LP1	45%	45%	10%	1:2	70%	30%	–	–
MR-LP2	45%	45%	10%	1:2	50%	50%	–	–
MR-LP3	45%	45%	10%	1:2	30%	70%	–	–
MR-MP1	45%	45%	10%	1:2	70%	–	–	30%
MR-MP2	45%	45%	10%	1:2	50%	–	–	50%
MR-MP3	45%	45%	10%	1:2	30%	–	–	70%
MR-BP1	45%	45%	10%	1:2	70%	–	30%	–
MR-BP2	45%	45%	10%	1:2	50%	–	50%	–
MR-BP3	45%	45%	10%	1:2	30%	–	70%	–

## Data Availability

The original contributions presented in this study are included in the article. Further inquiries can be directed to the corresponding author.
